# Cardiopulmonary resuscitation (CPR) complications encountered in forensic autopsy cases

**DOI:** 10.1186/s12873-019-0234-5

**Published:** 2019-02-28

**Authors:** Aspasia Deliliga, Fotios Chatzinikolaou, Dimitrios Koutsoukis, Ioannis Chrysovergis, Polychronis Voultsos

**Affiliations:** Department of Forensic Medicine & Toxicology, School of Medicine, Aristotle University of Thessaloniki, (Campus), School of Medicine, 54124 Thessaloniki, Greece

**Keywords:** Cardiopulmonary resuscitation, (un) avoidable CPR-related complications, Rib fractures, Sternal fractures

## Abstract

**Background:**

Cardiopulmonary resuscitation (CPR) provides a significant increase in survival rate, even if performed by bystanders. However, bystanders may refrain from performing CPR for fear of eventual malpractice litigation. Currently lack Guidelines specifying whether a particular CPR-related complication is in all likelihood unavoidable or not. To fulfill this gap a great number of studies is required to be published in the most relevant leading academic literature. This paper aims at making a contribution to addressing such a challenge.

**Methods:**

A retrospective observational study based on forensic autopsy material aiming at recording injuries resulting from the application of CPR. The severity of injuries was forensically evaluated.

**Results:**

Out of 88 cases autopsied, only 26.7% had rib fractures (only 20% of which were located in the 6 lower ribs), 17.4% had sternal fractures (85.7% of which were detected in the body of the sternum and 14.3% in the manubrium). The ratio of sternal fractures to rib fractures is similar to the ratio cited in other studies reported in the literature (2:3, approximately). The number of fractures was 7.86 (4.11 on the right side and 4.75 on the left side). 16% of the cases were found to be mild, 48% were moderate, and 35% of the cases were severe. When a physician was present, a (not statistically significant) trend towards more severe complications was found.

**Conclusion:**

The findings are in accordance with other similar studies reported in the literature referring to the classic external CPR. This study offers a proposal aiming at making a contribution to develop Guidelines specifying whether a particular CPR-related complication is in all likelihood unavoidable or not.

## Background

Cardiopulmonary Resuscitation (CPR) constitutes one of the most important interventions at the socio-medical level. It is a simple procedure which, however, may result in significant increase of the rate of survival of patients who experience sudden cardiac arrest mostly due to coronary heart disease [1].

In case of cardiac arrest resuscitation attempts should continue until adequate spontaneous circulation is achieved or until the death of the patient is ascertained [2]. Nevertheless, these attempts may cause considerable injuries to patients, thus increasing rescuer’s exposure to legal risk [2].

The injuries associated with the application of CPR are classified according to their incidence [2] and localization [3–6]. Interestingly, there are several publications reporting rare complications of CPR concerning both adults and minors [7–14].

According to the official website of the American Heart Association (AHA) [15], cardiac arrest is more frequent than the general public considers, and it may happen to anyone and at any time. Therefore, CPR training should not only be worthy of medical and paramedical staff’s concern, but also it should be considered worthy of anyone’s concern. According to the same source, four out of every five cases of cardiac arrest occur at home; thus, it is probable that a potentially life-saving CPR would be performed by a family member [15].

Despite the fact that the potential beneficial effect of CPR has been established, less than one out of three victims of out-of-hospital cardiac arrest receives life-saving assistance from a bystander [16].

CPR has been described as “violent, painful and undignified” [17]. However, it must be applied to all patients since it provides a 4-fold increase in survival rate, even if it is performed by bystanders [1].

Although out-of-hospital cardiopulmonary resuscitation (CPR) may result in a significant increase in survival rate even if it is performed by bystanders, certain CPR-related complications are unavoidable and, hence, bystanders in all likelihood may refrain from performing CPR for fear of eventual malpractice litigation even in jurisdictions where Samaritan laws are present. Currently lack Guidelines specifying whether a particular CPR-related complication is in all likelihood unavoidable or not. To fulfill this gap a great number of studies is required to be published in the most relevant leading academic literature. This paper aims at making a contribution to address such a challenge.

## Methods

### Study design

The purpose of this study was to record the incidence, localization, and severity of complications of CPR among individuals who did not survive their cardiac arrest in Thessaloniki (the second largest city in Greece) and associate them with the conditions under which CPR was performed, on the basis of our forensic autopsy findings. Precise assessment of the complications of CPR can only be made using forensic autopsy, since in most cases these injuries go radiologically undiagnosed [18]. This is the first study based on forensic autopsy material coming from Greece aiming at recording injuries resulting from the application of cardiopulmonary resuscitation. All the victims of cardiac arrest included in our study had undergone manual cardiopulmonary resuscitation. No one of them had undergone CPR including a mechanical chest compression device.

The records of 184 autopsies carried out in the Laboratory of Forensic Medicine and Toxicology of Aristotle University of Thessaloniki during a 12-month period in 2013 were examined. The study included all cases of violent and sudden deaths where CPR was performed.

Overall, 88 cases were included in the study. Our data involved demographic data, cause of death, type of death, number and skillfulness or experience of persons who performed CPR, presence and localization of soft tissue injuries, presence and localization of rib and sternal fractures, medications received, and location where CPR was performed. In cases where injuries due to CPR were identified, their severity was estimated forensically. Characterization of a category as “severe” neither corresponds to the “severe bodily injury” described in the Greek Penal Code (article 310), which refers to a life-endangering injury, nor to any other similar distinctions found in the international literature (e.g., at least 14 day hospitalization, or significant disability for at least 28 days) [19]. The injuries were classified into three categories (mild, moderate, severe) based on localization (proximity to the heart), number of fractured ribs (more or less than 6), history of medication interfering with the coagulation mechanism (factor which is also taken into consideration in other similar studies), and medical history, i.e., factors increasing the risk of complications. Whenever the number of fractures (with or without bleeding) was less than 6, injuries were considered mild. When the number of fractures ranged from 7 to 12, injuries were considered moderate, and when it ranged from 13 to 24, injuries were considered severe. The standard of “six fractured ribs” might be viewed as an arbitrary criterion, provided that the criteria used for classification of chest injuries depend on the criterion of 3 fractured ribs and on the clinical picture of the so-called “flail chest” (Open injury scaling, Chest Wall) [20], which, however, is absent in our necrotomic matter. Indeed, the classification into three categories (mild, moderate, severe) is not based on «gold standard». However, in our opinion a severity criterion necessarily involves a degree of arbitrariness. For instance, such a criterion should take into account both the number and the location of the injuries, provided that one only rib fracture located on the left half thorax and close to the heart may most likely lacerate it, even though, a greater number of rib fractures located on the right half thorax might be regarded as life-threatening injuries to a much lesser extent. We set our severity standards based on our forensic evaluation.

### Statistical analysis

Values are expressed as mean ± standard deviation (SD) or percentages. The independent samples t-test was employed for the comparison of means between groups. Pearson’s chi-squared test was used to check the independence between categorical variables. Logistic regression was used for the assessment of the impact of age on the occurrence of rib fractures. *P* values less than 0.05 were considered statistically significant. Statistical analysis was performed using SPSS v21.0 (IBM Corp, Armonk, NY). One-way analysis of variance was used to check differences between means of age for the different categories of injury severity.

## Results

The bodies were those of 53 males and 35 females. At the time of death the age range was 18–87 years (60.6 ± 7.5 years). Cardiovascular diseases were the most common cause of death (*n* = 62) and most deaths were non-violent (*n* = 76). There was no chest injury prior to the cardiac arrest in none of the cases. All the identified injuries were due to performance of CPR.

### Incidence and location of the injuries

All the CPR-related rib fractures involved in our autopsy findingswere located in (or very close to) the midclavicular line. As regards the CPR-related sternal fractures, 85.7% of these injuries were identified in the body of the sternum and 14.3% in the manubrium.

More specifically, out of 88 cases, only 26.1% had rib fractures. Most of them were located in the six upper ribs. The most common location of the fractures was the 2nd rib (in 39% of them bilaterally). In 8,6% of them a fracture of the 1st rib was co-existed. Moreover, 17.4% were accompanied with sternal fractures. Most of them were located in the body of the sternum (85.7%). Results are summarized in Table 1.

**Table 1:** Rib fractures (RF) location in association with sternal fractures (SF)

Right side RF	Left side RF	Sternal fractures (SF)	Incidence	N
✓	✓	✓	37%	10
✓	✓	–	29,6%	8
✓	–	–	7,4%	2
–	✓	–	7,4%	2
–	✓	✓	3,7%	1
–	–	✓	14,8%	4

As regards soft tissue injuries, 18.2% of cases were accompanied with soft tissue injuries, mostly identified on the left side (Table 2).

**Table 2:** Soft tissue injuries

Right side	Left side	Incidence	N
✓	–	1,2%	1
–	✓	10,6%	11
✓	✓	7,1%	6

A history of osteoporosis was only found in one case; however, no CPR-related injuries were identified.

All cases with sternal fractures referred to a single fracture.

### Statistical correlations

Statistical analysis showed independence between gender and rib or sternal fractures (*p* > 0.999 and *p* = 0.819, respectively).

Similarly, no correlation between age and rib or sternal fractures was found (*p* = 0.290 and *p* = 0.533 respectively).

### Who performed CPR and where

In 96.6% of the cases, CPR was performed by specialized staff (medical, paramedical or both), and in 2% of the cases it was performed by both specialists and laypersons. As regards the specialized staff, in 52.3% of the cases it was paramedical staff, in about 20% of the cases it was medical staff, while a coexistence of medical and paramedical staff was reported in 28% of the cases, approximately. In 29.1% of the cases, more than one person actively participated in the procedure (see Table 3). A physician was present in 47.7% of the cases. More severe injuries were more often found in cases where CPR was performed in the presence of a physician and in presence of more than one other person, although without statistical significance (*p* = 0.59 and 0 + .195, respectively). Whenever a physician was present, injuries were moderate or severe at equal frequency (see below), but they were never mild. In addition, in 58% of cases where CPR was performed in the presence of a physician, there was a second person present in the area as well, which complicates the interpretation of this finding. It is possible that the trend towards more severe injuries in the presence of a physician is either due to a more intensive application of CPR by the physician, or to a repetitive application of CPR (e.g., the physician arrives at the area at a later stage and repeats the procedure).

**Table 3:** Number of rescuers

		Frequency	Percent	Valid Percent	Cumulative Percent
Valid	1 Person	61	69,3	70,9	70,9
	> 1 Persons	25	28,4	29,1	100,0
	Total	86	97,7	100,0	
Missing	99	2	2,3		
Total		88	100,0		

The place where CPR was performed was the victim’s home in 27.3% of the cases, a public place in 22.7%, and a medical environment in 46.6% of the cases. This means that, in 50% of the cases, CPR was performed in out-of-hospital settings.

### The severity of injuries

None of the identified CPR-related injuries was lethal. The severity of injuries was evaluated in the aforementioned cases and it was found that 16% of them were mild, 48% were moderate, and 35% of the cases were severe. When CPR was performed by more than one person, the injuries were moderate or severe at an equal rate and they were never mild, whereas when it was performed by a single person, in 23.8% of the cases it resulted in mild injuries, in 47.6% it resulted in moderate injuries, and in 28.6% it caused severe injuries (See Table 4). Statistical analysis showed no association between severity of injuries and gender (chi-square = 1.022, df = 3, *p* = 0.796) (see Table 5 and Table 6). In addition, it was established that there is some independence between severity of injuries and type of staff (chi-square = 7.398, df = 6, *p* = 0.286) and also between severity of injuries and place where CPR was performed (chi-square = 8.614, df = 9, *p* = 0.474).

**Table 4:** Number of rescuers and evaluation of injuries severity

			Rescuers number		Total
			1 person	> 1 persons	
FForensic Evaluation of Severity	Mild	Count	5	0	5
		Percent	23,8%	,0%	16,1%
	Moderate	Count	10	5	15
		Percent	47,6%	50,0%	48,4%
	Severe	Count	6	5	11
		Percent	28,6%	50,0%	35,5%
TTotal		Count	21	10	31
		Percent	100,0%	100,0%	100,0%

**Table 5:** Gender and rib fractures (RF)

	Cases					
	Valid		Missing		Total	
	N	Percent	N	Percent	N	Percent
Gender of victims with RF	86	97,7%	2	2,3%	88	100,0%

**Table 6:** Gender and rib fractures (RF)

		Rib fractures		Total
		Yes	No	
Gender	Male	14	37	51
	Female	9	26	35
Total		23	63	86

## Discussion

The major finding of this study were that the incidence of rib and sternal fractures and of soft tissue injuries caused by CPR is consistent with the corresponding rates of other studies [3, 5, 6, 18] The ratio of rib to sternal fractures was similar to the ratio cited in other studies [3, 5, 6, 18].

A wide range of incidence rates of rib fractures caused by CPR (13–97%) [21] has been reported in the literature. Sternal fractures are reported at a rate usually ranging from 14 [3] to 30% [22] even though frequencies as low as 1% and as high as 43% are also reported [21]. Lederer et al. reported rib and sternal fractures at a rate of 94.7% following a successful CPR procedure [18]. In a forensic research conducted by the University of Edinburgh, which included 499 cases, Black et al. reported rib fractures in 29% of the cases (concerned mainly females) and sternal fractures in 14% of the cases [3]. The incidence of sternal fractures reported by various authors is almost by half compared to the reported incidence of rib fractures [3]. Rib fracture incidence was positively associated with advanced age. In 97 cases, where there were “multiple” rib fractures, no significant difference was found between right (4 ribs) and left side (4 ribs) [3]. In our study the average fractures were 7.86 overall (4.11 on the right side and 4.75 on the left side). A Turkish study (University of Pamukkale) including 231 deaths, reported an incidence rate of 13.2% for rib and sternal fractures [6]. Rabl et al. report an incidence rate of 28% for rib fractures and 16% for sternal fractures [23]. Similarly, Baubin et al. reported incidence rates of 55 and 30% [22], and Black et al. reported incidence rates of 29 and 14%, respectively [3]. Out of 96 autopsies overall, Hashimoto et al. reported incidence rates of 52% (43% on the right side, 48% on the left side, and 39% bilaterally) and 39%, respectively (only 9% of which was not accompanied by rib fractures) [5]. The average fractured ribs were 7.3 depending on the case. Hoke and Chamberlain report incidence rates of 13–97% and 1–43%, respectively, while they report almost zero incidence rates for children [21]. It is maintained in the literature that females have sternal fractures more frequently (since they have smaller and thinner sternum) [24], as well as that the elderly are at higher risk for rib fractures [25]. The incidence of rib fractures increases with age. However, this is not the case with sternal fractures [3]. Both rib and sternal fractures are rare among persons under 20 years of age [5]. Rib and sternal fractures are reported far more rarely in infants and children. Rib fractures often occur at the 3rd, 4th, or 5th rib, located in the midclavicular line of the rib cage [4]. However, in 60% of our cases with rib fractures was found a fracture of the 2nd rib.

Accordingly, sternal fractures occur most frequently in the region between the level of 3rd and 4th rib, or between the level of 4th and 5th rib [4]. Interestingly, Krischer et al. maintain that fractures in these regions might be considered unavoidable [4]. In 20% of their cases, they identified some avoidable fractures (at very high or very low ribs, at a rate of 5% at both high and low ribs). They report a rate of 4% for sternal fractures. A high incidence rate of fractures at the first and, mostly, at the second rib, even bilaterally, was found in our study. In no one of our case was found other CPR-related complication such as pericardial, pulmonary, cardiac trauma or thrombosis. Visceral injuries as well as dangerous air embolism after cannulation of the external jugular vein are considered rare complications. In addition, bone marrow embolism may be seen in peripheral vessels. It is reported in 13% of the cases [5].

The results of our study are consistent with the findings of other studies showing that there is no difference in the incidence rate of adverse effects when comparing a specialized staff with laypersons [26]. However, Kim et al. showed that the presence of non-physician chest compressors in the Emergency Department was one of the contributing factors to the formation of rib fractures [27].

The here presented findings might contribute on the enrichment of literature concerning CPR-related rib and sterna fractures. Interestingly, the greater the number of cases in literature with CPR-related rib and sternal fractures, the more reliable the consideration that certain CPR-related rib or sternal fractures were unavoidable, namely, should normally not raise malpractice litigation. Not surprisingly, it has been established that the adequate depth of chest compression is a matter of the utmost importance for an increase in the possibility of achieving spontaneous circulation. The European Resuscitation Council (ERC) has changed their recommendation about minimal compression depth from 40 mm (2005) to 50 mm (2010) [28, 29]. The quality of chest compressions has improved significantly after the 2010 AHA guidelines; however, it is more difficult for the rescuer to comply with the guidelines because of increased fatigue in long duration CPR [30].

According to Hellevuo et al. iatrogenic injuries increase as compression depth exceeds 6 cm, especially in case of male patient. However, it is worth noting that the injuries are characterized “by and large not fatal” [31].

Below, we go into some details concerning eventual CPR-related liability. It is widely accepted that when performing CPR, certain complications are ordinary and unavoidable, even if CPR was performed *lege artis* and with the due diligence. The physician performing CPR is aware of these complications and accepts their occurrence as necessary or, at least, as potential. This means ─ at least according to Greek and German Criminal Law ─ that there is some indirect “intent” (Intend Second Degree, namely, the physician knows that a certain incidental-perhaps undesired- consequence will occur) or conditional intent (Intend Third Degree, namely, the physician foresees the result as possible and accept it, albeit undesired) [32, 33]. Other complications, however, may be (fully or partially) due to an incorrect medical procedure (e.g., incorrect placing of the hands on the chest, excessive pressure force, etc.). Such complications are hard to distinguish from unavoidable complications. Such distinction is still immature both in theory/legal doctrine and in case-law. Nevertheless, such distinction is of vital importance whenever an issue of medical liability arises. The characterization of any complications as unavoidable may discharge a physician from liability. Either in the context of the existing negligence-based legal system or in an alternative (reform) legal system, e.g. a “no-fault” compensation system [19]. Under the Greek legal framework both civil and criminal liability may arise for a physician who has performed CPR. Article 29 of the Greek Penal Code (PC) along with article 310§1 PC provide for a sentence for a severe unintentional bodily injury whose outcome is due to negligence (of rescuer in the instance of CPR).

Not surprisingly, there is a “grey zone” between avoidable and unavoidable CPR complications. However, there is a “core” of complications which may be considered “unavoidable” almost with certainty. Such complications can be determined based on forensic studies which promise more reliable results and involve a larger number of cases provided that spontaneous circulation and hemodynamic stability is attainable in only a small number of cases.

Our study is an attempt to contribute to determining the rib and sternum CPR-related injuries that might be viewed as “in all likelihood unavoidable”. Only classic osseous injuries of the ribs and sternum were identified at a frequency similar to that reported in other studies, i.e. as regards their locations. There was no correlation with factors such as gender, age, place where CPR is performed, or rescuer. This conclusion is considered to favour the classification of particular bone injuries among the unavoidable complications. However, it is crucial to bear in mind that it would be a difficult task to accurately determine the incidence of CPR-related rib and sternal fractures. Further research is needed in this perspective. Miller et al. arguably state that “the incidence of reported CPR-associated cardiovascular and thoracic wall injuries varies widely” [34]. The authors put it best in saying that “this may reflect several factors including the quality of the chest compressions and CPR, the diligence in defining these complications in survivors and non-survivors and the varying sensitivity of different diagnostic modalities to detect these injuries” [34]. Takayama et al. recently argue that “long duration of out-of-hospital CPR was an independent risk factor for chest injuries, possibly due to the difficulty of maintaining adequate quality of CPR.” [35]. Kralj et al. showed that “increased compression rate and depth cause more skeletal chest injuries (SCI)” [36]. Although “it is generally considered that at least 1/3 of resuscitated patients sustain rib fractures and at least 1/5 sustains sternum fractures”, Kralj et al. showed that these injuries are much more frequent [36].

## Conclusion

In accordance with other similar studies reported in the literature referring to the classic external CPR it is considered that rib and sternal fractures located at the 3rd, 4th, and 5th ribs and at the corresponding part of the body of the sternum, not extending beyond the midclavicular line of the rib cage, might possibly be described as unavoidable injuries. This consideration presupposes that the average rib fractures should not exceed 7 or 8, with almost equal bilateral distribution (with a slight deviation for the left side). Besides, the ratio sternal fractures: rib fractures should be equal to the fraction 2/3 (approximately).

### Proposition

In our opinion, the findings of our study which are in accordance with the available relevant literature encourage further research in the perspective of confirming the following Guideline:

In case of adult patients, the following complications might be classified as unavoidable:

A) Rib fractures (of 3rd, 4th, or 5th rib) which do not extend beyond the midclavicular line of the rib cage;
B) The total number of which does not exceed 6 fractures;
C) There is almost equal distribution of right- and left-sided rib fractures, with a slight deviation for the left side;
D) There is one or two sternal fractures at a level corresponding to the 3rd-5th rib.

Note, however, that osteoporosis or any other anatomical variation of the chest should not be present. These are elements that must be considered in the forensic medical examination on a case-by-case basis.

It is also suggested that more rigorous forensic investigations (involving the consideration of the detailed anatomical and histological variations of any given chest wall, eventually using ad hoc models of CPR) is needed for greater certainty on the location and the number of fractures that might be regarded as “in all likelihood unavoidable complications”.

Our proposal aims at making a contribution to develop Guidelines specifying with high degree of certainty whether a particular CPR-related complication is in all likelihood unavoidable or not.

These Guidelines are expected to prevent potential rescuers from practicing “negative defensive medicine” in the context of emergency medicine in terms of avoiding the performance of CRP due to fear of malpractice litigation. At any rate, it is crucial to bear in mind that those who provide emergency medical service should be secured “potential to provide better public service” [37].

### Limitations

We have no data on CPR duration. Provided that the longer the duration of CPR was, the greater the number of CPR-related complications might be, lack of data concerning the duration of CPR is a limitation of our study. Another limitation of our study is that we have no data concerning the gender of the rescuers.

## Abbreviations

AHA: American Heart Association
CPR: Cardiopulmonary Resuscitation
ERC: European Resuscitation Council
PC: Penal Code
